# Exploring the Immunomodulatory Potential of Pancreatic Cancer-Derived Extracellular Vesicles through Proteomic and Functional Analyses

**DOI:** 10.3390/cancers16101795

**Published:** 2024-05-08

**Authors:** Anna Piro, Maria Concetta Cufaro, Paola Lanuti, Davide Brocco, Laura De Lellis, Rosalba Florio, Serena Pilato, Sara Pagotto, Simone De Fabritiis, Simone Vespa, Giulia Catitti, Fabio Verginelli, Pasquale Simeone, Damiana Pieragostino, Piero Del Boccio, Antonella Fontana, Antonino Grassadonia, Mauro Di Ianni, Alessandro Cama, Serena Veschi

**Affiliations:** 1 Department of Pharmacy, G. d’Annunzio University of Chieti-Pescara, 66100 Chieti, Italy; anna.piro@unich.it (A.P.);; 2 Center for Advanced Studies and Technology (CAST), G. d’Annunzio University of Chieti-Pescara, 66100 Chieti, Italy; 3 Department of Medicine and Aging Sciences, G. d’Annunzio University of Chieti-Pescara, 66100 Chieti, Italy; 4 Department of Medical, Oral and Biotechnological Sciences, G. d’Annunzio University of Chieti-Pescara, 66100 Chieti, Italy; 5 UdA–TechLab, Research Center, G. d’Annunzio University of Chieti-Pescara, Via dei Vestini, 66100 Chieti, Italy; 6 Department of Innovative Technologies in Medicine and Odontoiatry, G. d’Annunzio University of Chieti-Pescara, 66100 Chieti, Italy; 7 Hematology Unit, Department of Oncology and Hematology, Santo Spirito Hospital, 65124 Pescara, Italy

**Keywords:** pancreatic cancer, extracellular vesicles, immune response

## Abstract

**Simple Summary:**

Pancreatic cancer (PC) develops resistance to current therapeutic approaches with a consequent dismal prognosis. Immunotherapy is an effective approach in several tumors, but PC is resistant to immunotherapy. Tumor-derived extracellular vesicles (EVs) may modulate immune responses by either dampening or inducing antitumor immune responses. In this study, we explored the immunomodulatory potential of pancreatic-cancer-derived extracellular vesicles through proteomic and functional approaches. Notably, proteins involved in the “Immune System” were highly enriched in the protein cargo of PC-derived EVs, which also included immunostimulatory proteins. Interestingly, the treatment of healthy donor-derived peripheral blood mononuclear cells (PBMCs) with EVs from one of the PC cell lines analyzed induced early activation markers in CD8+ and CD4+ lymphocytes. This was consistent with the proteomic and ELISA analyses. Our study indicates that even if PC is an immune-cold tumor, in some cases, PC-EVs may activate early immune responses. This finding might be relevant for the development of effective immunotherapeutic strategies in PC.

**Abstract:**

Pancreatic cancer (PC) has a poor prognosis and displays resistance to immunotherapy. A better understanding of tumor-derived extracellular vesicle (EV) effects on immune responses might contribute to improved immunotherapy. EVs derived from Capan-2 and BxPC-3 PC cells isolated by ultracentrifugation were characterized by atomic force microscopy, Western blot (WB), nanoparticle tracking analysis, and label-free proteomics. Fresh PBMCs from healthy donors were treated with PC- or control-derived heterologous EVs, followed by flow cytometry analysis of CD8+ and CD4+ lymphocytes. The proteomics of lymphocytes sorted from EV-treated or untreated PBMCs was performed, and the IFN-γ concentration was measured by ELISA. Notably, most of the proteins identified in Capan-2 and BxPC-3 EVs by the proteomic analysis were connected in a single functional network (*p* = 1 × 10^−16^) and were involved in the “Immune System” (FDR: 1.10 × 10^−24^ and 3.69 × 10^−19^, respectively). Interestingly, the treatment of healthy donor-derived PBMCs with Capan-2 EVs but not with BxPC-3 EVs or heterologous control EVs induced early activation of CD8+ and CD4+ lymphocytes. The proteomics of lymphocytes sorted from EV-treated PBMCs was consistent with their activation by Capan-2 EVs, indicating IFN-γ among the major upstream regulators, as confirmed by ELISA. The proteomic and functional analyses indicate that PC-EVs have pleiotropic effects, and some may activate early immune responses, which might be relevant for the development of highly needed immunotherapeutic strategies in this immune-cold tumor.

## 1. Introduction

Pancreatic cancer (PC) is one of the most aggressive and lethal cancers, with a dismal prognosis. Survival rates for PC patients are among the lowest for solid tumors, with a 5-year survival rate of about 10% [1]. PC exhibits limited or no response to chemotherapy and radiotherapy and is resistant to immunotherapy, although this therapeutic approach has shown promising results for various malignant tumors [2]. PC is a poorly immunogenic tumor characterized by an immunosuppressive microenvironment marked by an imbalance in immune cell populations. Among others, immunosuppressive T regulatory cells (Tregs), M2 polarized tumor-associated macrophages, and myeloid-derived suppressor cells (MDSCs) are more abundant compared to both cytotoxic CD8 T and dendritic cells [1]. Extracellular vesicles (EVs) are released by almost all cell types in various biological fluids and cell culture supernatants [3,4]. They transfer precise biological signals to recipient cells, serving as vital regulators of structured cell communities in various physiological and pathological processes, including cancer [3,4,5]. In particular, EVs are involved in the initiation, progression, and metastasis of different types of tumors and mechanisms related to tumor microenvironment (TME) remodeling [4,6]. EVs have also emerged as significant regulators of the immune system, with the potential to inhibit or activate immune responses [7]. Several studies analyzed the role of tumor-derived small EVs and found that they may directly suppress the proliferation and activation of CD8+ T cells [8,9]. Moreover, EVs favor immunosuppressive TME by inducing the differentiation of monocytes into MDSCs and stimulating the expansion of Tregs, thus causing suppression of effector T cells [9]. In addition, tumor-derived EVs promote polarization of macrophages into an M2-like phenotype, contributing to an environment that supports tumor growth, angiogenesis, and tissue remodeling [10]. Also, PC-derived EVs were found to suppress immune responses by regulating various immune cells and processes [2,11]. In particular, it was reported that the uptake of PC-derived exosomes by isolated T lymphocytes may induce ER stress-mediated apoptosis of pre-stimulated T lymphocytes [12]. Moreover, PC-EVs compromise DC antigen presentation, thereby impairing their ability to activate CD4+ T cells, promote the expansion of suppressor MDSCs in the TME, and polarize macrophages into the M2 phenotype [2,13,14]. Intriguingly, opposite to their immunosuppressive activity, EVs derived from several malignancies, such as T-cell lymphoma, melanoma, and colon carcinoma, were shown to induce the immune activation of NK antitumor–cytotoxic activity and activate dendritic cells, thereby priming the immune system to kill cancer cells [15,16,17]. Notably, cancer EVs can present tumor antigens to dendritic cells, thereby triggering the activation of T cells that inhibit tumor progression [18,19]. In fact, exosomes derived from colon carcinoma, mammary carcinoma, mesothelioma, mastocytoma, and chronic myeloid leukemia were used to load dendritic cells, triggering T-cell-mediated antitumor immune responses, which led to rejections of autologous tumors and strong inter-tumor cross-protection in mice [19]. Notably, not all tumor-derived exosomes were effective in triggering antitumor immune responses [19]. At present, most studies on EVs derived from pancreatic cancer cells focus on their immunosuppressive potential, whereas it is unknown whether PC-derived EVs, in their native state, also possess an intrinsic immune-stimulatory potential. In this regard, a previous study showed that EVs derived from the PANC-1 cell line, after the depletion of miRNA cargo, had immunostimulatory effects. After this manipulation, PANC-1-derived EVs gained a dendritic-cell-mediated immune stimulatory effect on cytokine-induced killer cells (CIKs), greater than unmanipulated EVs and comparable to that obtained by stimulating dendritic cells with LPS [20]. However, the intrinsic stimulatory activity of unmanipulated PC-derived EVs was not evaluated in that study. The immunomodulatory role of PC-derived EVs on T lymphocytes within the context of peripheral blood mononuclear cells (PBMCs) has not been explored before. Considering that this experimental condition may reflect the physiological conditions where T lymphocytes can interact with costimulatory cells [21], we analyzed the effect of PC-derived EVs in this context. Using multiple approaches, including the proteomic profiling of PC-derived EVs and the treatment of PBMCs with these EVs, followed by the functional and proteomic analyses of CD3 lymphocytes, we observed that PC-derived EVs expressed immunomodulatory proteins and had immunostimulatory or no effect on CD4+ and CD8+ lymphocytes, depending on the cell line from which they were derived.

## 2. Materials and Methods

### 2.1. Cell Line and Cultures

The human pancreatic cancer (PC) cell lines Capan-2 and BxPC-3 were purchased from Cell Lines Service ATCC (American Type Culture Collection, Manassas, VA, USA). The PC cell lines were cultured in RPMI 1640 supplemented with 10% fetal bovine serum, 1% Pen/Strep, and 1% L-glutamine (Sigma-Aldrich, Saint Louis, MO, USA). To obtain PC extracellular vesicles (EVs) from Capan-2 or BxPC-3, the cells (8 × 10^6^) were seeded in T175 cell culture flasks, and the following day, the media was replaced with RPMI 1640 supplemented with exosome-depleted FBS by ultracentrifugation, according to the Beckman Coulter protocol. The culture media for EV isolation were collected after 72 h.

### 2.2. EV Isolation

The extracellular vesicles (EVs) from conditioned media were isolated by sequential centrifugation and ultracentrifugation, essentially according to the protocol previously described by Théry, with some modifications [22]. Briefly, the initial procedures were executed to remove large dead cells and cellular debris through sequential centrifugation at escalating speeds. During each step, the pellet was discarded, and the supernatant was utilized for the subsequent stage. The ultimate supernatant underwent ultracentrifugation at 100,000× *g* to precipitate EVs. [22]. EV pellets were resuspended in 100–200 µL of RPMI 1640 with exosome-depleted FBS.

### 2.3. EV Characterization

#### 2.3.1. Nanoparticle Tracking Analysis (NTA)

The size of EVs was determined by a ZetaView instrument (Particle Metrix GmbH, Ammersee, Germany), which is equipped with fast video capture and nanoparticle tracking software.

#### 2.3.2. Atomic Force Microscopy Analysis (AFM)

In order to investigate the size distribution and morphology of the isolated EVs, Atomic Force Microscopy (AFM) analysis was performed using a MultiMode 8 AFM microscope with a Nanoscope V controller (Bruker, Billerica, MA, USA). The samples were prepared by depositing a drop of diluted suspensions of isolated vesicles on a SiO_2_ wafer, followed by drying in the oven at 37 °C for 2 h and then at room temperature overnight. These preparations were scanned by the silicon RTESPA-150 probe (rectangular geometry; cantilever resonance frequency, 150 kHz; and nominal spring constant, 5 N/m) in TappingMode™ in air. Images of 512 × 512 pixels were collected with different scan sizes of 1.5 µm^2^ and were elaborated using NanoScope Analysis 1.8 software (Bruker, Billerica, MA, USA).

#### 2.3.3. Western Blot Analysis

Cells and EVs were collected and lysed in RIPA buffer (Sigma, St. Louis, MO, USA) supplemented with a protease and phosphatase inhibitor cocktail and phenylmethanesulfonyl fluoride (PMSF, Sigma, St. Louis, MO, USA). Protein concentrations were determined by the BCA Protein Assay (Thermo Scientific, Rockford, IL, USA), and 40 μg were subjected to electrophoresis, followed by immunoblotting, as previously described [23]. Mouse monoclonal CD81 and Cytochrome C antibodies were obtained from Santa Cruz Biotechnology, Inc. (Dallas, TX, USA). Mouse monoclonal CD63 and rabbit monoclonal Flotillin-1 antibodies were purchased from Invitrogen (Thermo Fisher Scientific, Waltham, MA, USA) and Cell Signaling Technology, Inc. (Beverly, MA, USA), respectively. Blots were revealed by chemiluminescence using Westar ηC Ultra 2.0 chemiluminescence substrate (Cyanagen, Bologna, Italy).

### 2.4. Peripheral Blood Mononuclear Cell (PBMC) Purification and Counts

Peripheral blood mononuclear cells (PBMCs) were isolated from the fresh whole blood of healthy donors by density gradient centrifugation using Ficoll-Paque PLUS (GE Healthcare, Chicago, IL, USA), as previously reported [24]. PBMCs were stained using 10 µL of 7-Amino Actinomycin D (7-AAD, Via-Probe, BD Biosciences, Cat. 555815, San Jose, CA, USA), and concentrations of viable PBMCs (7-AAD-events) were obtained by flow cytometry using a FACSVerse analyzer equipped with a volumetric count module (BD, Becton-Dickinson Biosciences, San Jose, CA, USA). All procedures involving human participants were carried out in accordance with the ethical standards of the 1964 Helsinki Declaration and its later amendments or with comparable ethical standards. This study was approved by the local ethics committee (V 1.0, 25 February 2016; V. 2.0, 21 January 2020). All human participants gave written informed consent.

### 2.5. Flow Cytometry Analysis

As described above, we treated fresh PBMCs of healthy donors with EVs derived from PC cells (Capan-2 or BxPC-3) or EVs derived from the CD3+ of healthy donors. After treatment, PBMCs were gathered together in PBS and stained at room temperature, shielded from light for 30 min using the mix outlined in Supplementary Table S1. Following labeling, the specimens underwent PBS washes via centrifugation at 1500 rpm for 10 min to remove excess antibodies. The gating strategy and the assessment of non-specific fluorescence were established by fluorescence minus one (FMO) control [25]. Data were acquired with a FACSVerse analyzer equipped with a volumetric count module (BD Biosciences, San Jose, CA, USA) and Cytoflex (Beckman Coulter, Brea, CA, USA).

### 2.6. Treatment of PBMCs with Capan-2 or Control Heterologous EVs and Fluorescence-Activated Cell Sorting of CD3+ Lymphocytes

Fresh PBMCs of healthy donors were seeded in 6-well plates (5 × 10^6^ cells/well) in RPMI 1640 supplemented with 10% exosome-depleted FBS (according to the Beckman Coulter protocol). Heterologous control EVs from healthy donors were freshly isolated after 24 h of culture by ultracentrifugation from the conditioned media of PBMCs, as described above for PC EVs. PBMCs from healthy donors were treated for 48 h with 150 μg of EVs/2 mL derived both from Capan-2 or heterologous control EVs. After EV treatment, PBMCs were stained using an allophycocyanin-conjugated anti-CD3 (3 μL), followed by washing and fixing with Cytofix/Cytoperm (BD Bioscience, San Jose, CA, USA, Cat. 554714) 1× for 15 min. CD3+ lymphocytes were then isolated (100 μm nozzle) by a fluorescence-activated cell sorter (FACS, FACSAria III, BD Biosciences) [26] and were subjected to a proteomic analysis. A purity of at least 90% was achieved for each isolated population.

### 2.7. Label-Free Proteomic Analysis

To evaluate the protein cargo of Capan-2- or BxPC-3-derived EVs, 30 μg of proteins from extracellular vesicle lysate was employed for proteomics investigation. A proteomic analysis was also conducted on CD3+ 1 × 10^6^ lymphocytes sorted from Capan-2-EV-treated PBMCs. Samples were prepared using the Filter-Aided Sample Preparation (FASP) protocol. In particular, for lymphocyte proteomics, they were lysed by sonication on ice (Sonicator U200S control, IKA Labortechnik, Staufen, Germany) at 70% amplitude in a lysis buffer (urea 6 M in 100 mM Tris/HCl, pH = 7.5). For both Capan-2- and BxPC-3-derived EVs and sorted CD3+ lymphocytes, overnight tryptic digestion was performed at 37 °C; then, tryptic peptides were analyzed in triplicate by LC-MS/MS using the UltiMateTM 3000 UPLC (Thermo Fisher Scientific, Milan, Italy) chromatographic system coupled to the Orbitrap FusionTM TribridTM (Thermo Fisher Scientific, Milan, Italy) mass spectrometer, following the analytical parameters detailed in Potenza et al. [27]. Briefly, the whole analysis was set at 65 min, with a chromatographic gradient from 2% to 90% of phase B (mobile phase A: H_2_O 0.1% formic acid, mobile phase B: acetonitrile 0.1% formic acid) acquiring positive-ion polarity with Data Dependent Acquisition (DDA) and HCD fragmentation by covering a *m*/*z* range of 300–1200. The MaxQuant version 1.6.10.50 (Max-Planck Institute for Biochemistry, Martinsried, Germany) was used for MS/MS raw data processing using Andromeda as an integrated search engine with the UniProt database (released 2020_06, taxonomy Homo Sapiens, 20,588 entries). The parameters and specifications for proteomic processing by the Perseus version 1.6.10.50 (Max-Planck Institute for Biochemistry, Martinsried, Germany) are described in our previous works [28,29,30]. For functional analysis performed by the Ingenuity Pathway Analysis tool (IPA, Qiagen, Hilden, Germany) [31], LFQ Intensities were used to quantify the protein abundance in each analyzed sample and uploaded into IPA as ratios (Capan-2-EV-treated/untreated CD3; Control-EV-treated/untreated CD3). Comparison Analysis was used to identify similarities or different trends between the various comparative proteomic results. Using IPA, we compared the upstream and downstream effects induced by Capan-2-derived EVs in CD3 (Capan-2-EV-treated/untreated CD3) versus those induced by heterologous control EVs (Control-EV-treated/untreated CD3). The pathway enrichment in the EV proteomic analysis was evaluated both by IPA and STRING (v. 12; https://string-db.org/, accessed on 1 May 2022). The biofunction expression analysis of Capan-2 and BxPC-3 EV protein signatures were graphed as bubble plots through the 2021–2023 SRplot free online platform [32].

### 2.8. Elisa Assay

INF-γ concentrations were measured in supernatants of EV-treated or untreated PBMCs by the Human INF-γ ELISA Kit, according to manufacturer instructions (Invitrogen by Thermo Fisher Scientific, Waltham, MA, USA). PBMCs were isolated from three different healthy donors. Photometric measurements were performed at a specific wavelength of 450 nm and 550 nm by the Infinite F50 absorbance plate reader (Tecan, Männedorf, Switzerland). INF-γ concentration values were obtained by interpolation with a standard curve created using standards at known and increasing concentrations of INF-γ.

### 2.9. Statistical Analysis

Statistical analyses were performed using GraphPad Prism version 5.01 software (San Diego, CA, USA). Comparisons of mean values were performed by an unpaired Student’s *t*-test. A *p*-value ≤ 0.05 was considered statistically significant (* *p* < 0.05; ** *p* < 0.01; *** *p* < 0.001; **** *p* < 0.0001).

## 3. Results

### 3.1. Characterization of EVs Derived from Pancreatic Cancer Cell Lines

**Figure 1:**
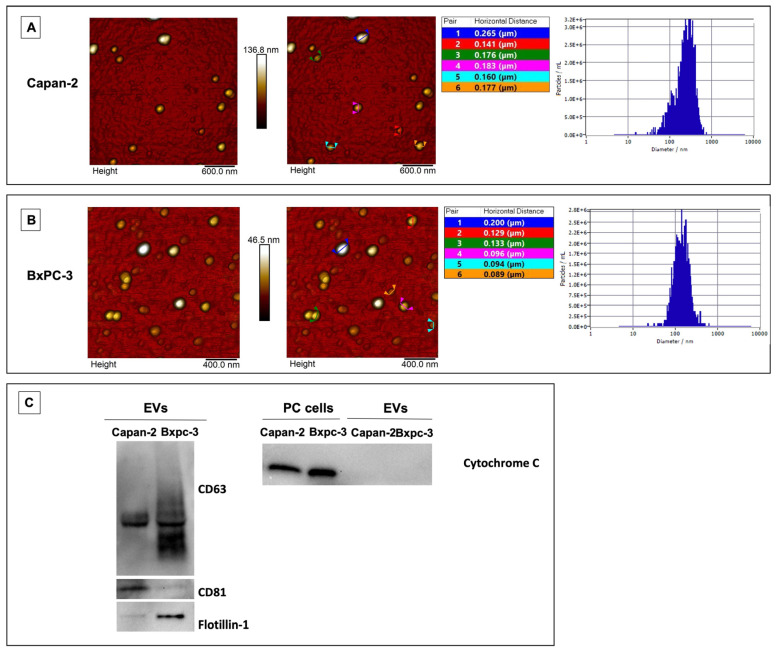
Characterization of Capan-2- and BXPC-3-derived EVs. Morphological features by AFM and sizing by NTA of EVs derived from Capan-2 (**A**) or BxPC-3 (**B**). Two-dimensional Atomic Force Microscopy topography images of EVs isolated from Capan-2 or BXPC-3 cells, with corresponding height cross-sectional profiles shown. In the tables, the horizontal distances related to each couple of colored markers are reported. On the right of panels A and B, nanoparticle tracking of size (diameter/nm) and concentration (particles/mL) of secreted vesicles produced by Capan-2 or BxPC-3 cells. According to NTA, EVs derived from Capan-2 or BxPC-3 showed an average diameter of 241 nm and 137 nm, respectively, falling within the size range detected by AFM. Western blot analysis of EV markers (**C**). To the left, representative Western blot of CD63, CD81, and Flotillin-1 in EVs derived from Capan-2 or BxPC-3. EVs expressed the three positive protein markers. To the right, representative Western blot of Cytochrome C, a negative protein marker of EVs, in whole-cell lysates of Capan-2 or BxPC-3 and their EVs. As expected, Cytochrome C was not present in vesicle fractions.

The human pancreatic cancer cell lines Capan-2 and BxPC-3 were cultured, and extracellular vesicles (EVs) were isolated by sequential centrifugation and ultracentrifugation. EVs derived from Capan-2 and BxPC-3 were characterized by Atomic Force Microscopy (AFM) for the evaluation of their size and integrity. Most of the EVs derived from the two PC cell lines showed a globular shape and were in an isolated form (Figure 1A,B). The diameter and size distribution of EVs were measured by Nanoparticle Tracking Analysis (NTA). According to NTA, the EVs derived from Capan-2 or BxPC-3 showed a median diameter of 241 nm and 137 nm, respectively, with a peculiar size distribution. In particular, the NTA peak analysis indicates that the diameter of Capan-2 EVs was 292 nm in 84.5%, 112 nm in 13%, and 67 nm in 2.6% of EVs, respectively, whereas the diameter of BxPC-3 EVs was 117 nm in 37.6%, 172 nm in 35%, and 142 nm 26.6% of EVs, respectively. The NTA results are in line with the size range detected by AFM for the EVs derived from the two PC cell lines (Figure 1A,B). EVs were further characterized by Western blot analysis for three positive protein markers of EVs and one EV negative protein marker, according to MISEV2018 and MISEV2023 guidelines [33,34]. EVs derived from Capan-2 or BXPC-3 expressed CD63 and CD81 tetraspanins and the cytosolic Flotillin-1 protein (Figure 1C), while Cytochrome C was exclusively detectable in the cell lysate of the PC cell lines and absent from EV fractions (Figure 1C). Cytochrome C is predominantly located within cell mitochondria, and it serves as a negative marker for EVs. Its detection in EV preparations implies potential contamination with cellular remnants like debris or apoptotic bodies. Thus, the absence of cytochrome C signals a clean EV sample devoid of organelle elements or apoptotic blebs, ensuring the integrity of the preparation.

### 3.2. Proteomic Analysis of Capan-2- and BxPC-3-Derived EVs

**Figure 2:**
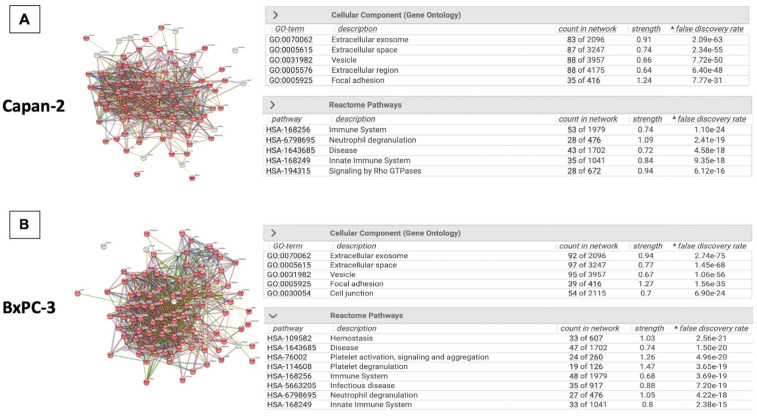
Protein expression analysis of EVs isolated from Capan-2 and BxPC-3 culture media. (**A**) The panel reports the STRING network of 95 proteins identified in Capan-2-derived EVs. The proteins are connected in a single functional network (PPI enrichment *p*-value < 1.0 × 10^−16^). Notably, 83 of the 95 identified proteins are involved in “Extracellular Exosome” (FDR 2.09 × 10^−63^), in line with the EV origin of the protein dataset, and 53 of the 95 identified proteins are involved in “Immune System” (FDR 1.10 × 10^−24^). (**B**) The panel reports the STRING network of 97 proteins identified in BXPC-3-derived EVs. The proteins are connected in a single functional network (PPI enrichment *p*-value < 1.0 × 10^−16^). Notably, 92 of the 97 identified proteins are involved in “Extracellular Exosome” (FDR 2.74 × 10^−75^), in line with the EV origin of the protein dataset, and 48 of the 97 identified proteins are involved in “Immune System” (FDR 3.69 × 10^−19^).

After the morphological and dimensional characterization of EVs derived from pancreatic cancer cell lines, we analyzed their protein cargo. The proteomic analysis of Capan-2 and BxPC-3 EVs identified 95 and 97 proteins, respectively (Supplementary Table S2). In both cases, these proteins were connected in a single functional network (*p* = 1 × 10^−16^) by STRING analysis (Figure 2A,B). According to STRING, 83 of the 95 proteins identified in Capan-2 were involved in the “Extracellular exosome” (GO Cellular component, FDR: 2.09 × 10^−63^), confirming the EV origin of the protein dataset (Figure 2A). Intriguingly, the majority of these proteins (*n* = 53/95) were involved in the “Immune System” pathway (Reactome Pathways, FDR: 1.10 × 10^−24^). Similarly, 92 of the 97 proteins identified in BxPC-3 were involved in the “Extracellular exosome” (GO Cellular component, FDR 2.74 × 10^−75^) (Figure 2B), and the majority of these proteins (*n* = 48/92) were involved in the “Immune System” pathway (Reactome Pathways, FDR: 3.69 × 10^−19^).

**Figure 3:**
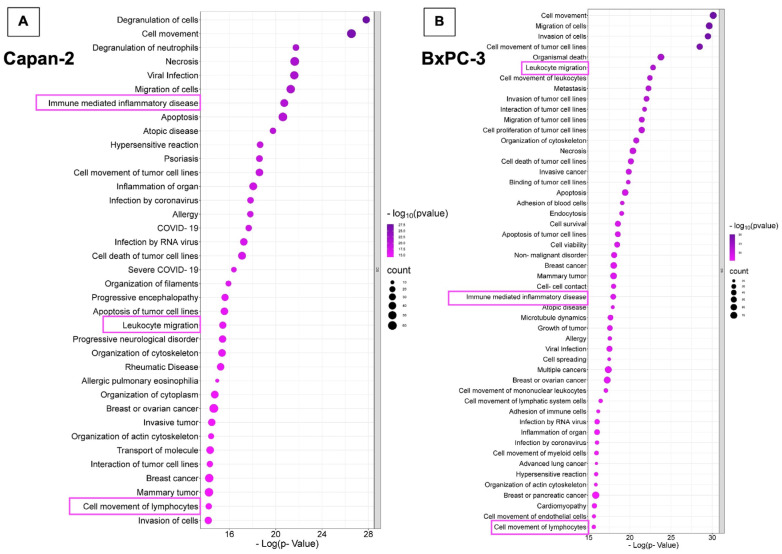
Functional assignments of Capan-2 and BxPC-3 EV proteins. The panel shows the functional assignments of Capan-2 (**A**) and BxPC-3 (**B**) EV proteins as bubble plots, where the counts and statistical significance of biological processes obtained with Ingenuity Pathway Analysis (IPA tool) are reported in the adjacent legends.

In line with the STRING analysis of proteins expressed in EVs isolated from Capan-2, “Immune mediated inflammatory disease” (*p* = 1.84 × 10^−21^), “Leukocyte migration” (*p* = 3.74 × 10^−16^), and “Cell movement of lymphocytes” (*p* = 6.10 × 10^−15^) were among the highest ranked IPA downstream pathways (Figure 3A and Supplementary Table S3). Similarly, the analysis of expressed proteins in BXPC-3-derived EVs showed that “Leukocyte migration” (*p* = 1.53 × 10^−23^), “Immune mediated inflammatory disease” (*p* = 1.05 × 10^−18^), and “Cell movement of lymphocytes” (*p* = 2.39 × 10^−16^) were among the highest ranked IPA downstream pathways (Figure 3B and Supplementary Table S3). Overall, the STRING and IPA analyses showed that cargo proteins of both Capan-2- and BXPC-3-derived EVs were highly enriched in proteins involved in the immune system response.

Notably, there were several differences in the EV protein cargo of the two cell lines with regard to proteins of the “immune pathway” identified in the STRING analysis (Supplementary Tables S4 and S5). In particular, 35 proteins were in common, and 32 were not shared between EVs derived from the two PC cell lines, with 19 proteins unique to Capan-2 and 13 proteins unique to BxPC-3 (Supplementary Table S5). In addition to these immunomodulatory proteins resulting from the STRING analysis, some of the proteins identified by the proteomic analysis in EVs derived from Capan-2 (Supplementary Table S2) are known to be antigens involved in immune activation, such as mesothelin, HSP90, and HSP70 [7]. HSP90 and HSP70, but not mesothelin, were also detected by the proteomic analysis in EVs derived from BxPC-3. Notably, this difference in mesothelin expression between EVs from these two lines is consistent with the drastic difference in mesothelin transcript expression between the two cell lines reported in The Human Protein Atlas (Capan-2: 497,5 nTPM, vs. BxPC-3: 87,1 nTPM; www.proteinatlas.org, accessed on 1 January 2024).

### 3.3. Capan-2-Derived EVs Increase the Expression of Early Activation Markers in CD4+ and CD8+ Lymphocytes

**Figure 4:**
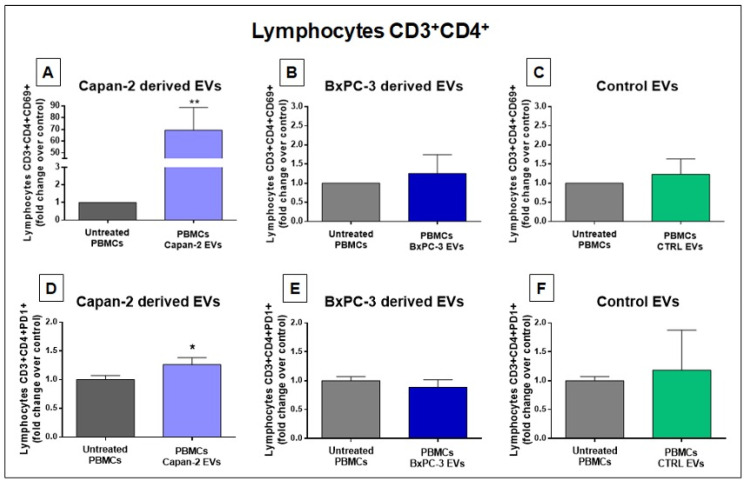
Capan-2, but not BxPC-3 or control EVs, increases the proportion of CD4+CD69+ and CD4+PD-1+ lymphocytes. The proportion of CD4+CD69+ lymphocytes was markedly increased after 48 h treatment of PBMCs with EVs derived from Capan-2 cell line (**A**) in comparison with untreated PBMCs, whereas treatment with BXPC-3-derived EVs (**B**) or heterologous control EVs (**C**) did not increase the proportion of CD4+CD69+ lymphocytes. The proportion of CD4+PD-1+ lymphocytes shows a slight increase only after treatment of PBMCs with EVs derived from Capan-2 cells (**D**) but not with BXPC-3-derived EVs (**E**) or heterologous control EVs (**F**). The graphed results are the mean + SD of at least three determinations, using PBMCs and heterologous EVs isolated from two different healthy donors. * Statistically significant differences between untreated PBMCs and EV-treated PBMCs (* *p* < 0.05, ** *p* < 0.01).

Considering that several pathways related to the immune system and immune modulation were among the highest ranked IPA downstream pathways according to STRING and IPA (Figure 2 and Figure 3), we analyzed the effect of PC EVs on the expression of CD69, a classical early marker of lymphocyte activation [35], and Programmed Death-1 (PD-1/CD279), another marker of early lymphocyte activation. In particular, we analyzed by flow cytometry CD69 and PD-1 expression in CD3+CD4+ and CD3+CD8+ cells derived from PBMCs untreated or treated for 48 h with EVs derived from PC cell lines (Capan-2 and BxPC-3). As a further control, we analyzed CD69 and PD-1 expression in CD3+CD4+ and CD3+CD8+ cells derived from PBMCs treated or untreated with heterologous EVs derived from unmatched healthy donors to verify whether heterologous EVs from non-cancerous cells could induce an effect on these markers. We studied these effects after EV stimulation of PBMCs because this experimental condition may reflect physiological conditions where T lymphocytes can interact with costimulatory cells. The analysis of CD4+ in PBMCs from two healthy donors treated with EVs derived from Capan-2 cells revealed that the proportion of CD3+CD4+CD69+ lymphocytes were markedly increased compared to untreated PBMCs (*p* = 0.0036) (Figure 4A). Conversely, in PBMCs from the same healthy donors, we did not observe significant changes in the proportion of CD3+CD4+CD69+ lymphocytes after PBMC treatment with BxPC-3-derived EVs or heterologous control EVs (Figure 4B,C). The results of flow cytometry analysis using Programmed Death-1 (PD-1/CD279) were consistent with those obtained with CD69. Namely, in healthy donor PBMCs, we observed a slight increase in the proportion of CD3+CD4+PD-1+ lymphocytes only after treatment with EVs derived from Capan-2 cells (*p* = 0.0326) (Figure 4D) but not with BxPC-3-derived EVs and heterologous control EVs (Figure 4E,F). These results with CD69 and PD-1 are in line with the activation of CD4+ T cells after a 48 h treatment with Capan-2-derived EVs but not after treatment with EVs from BxPC-3 or heterologous control EVs.

**Figure 5:**
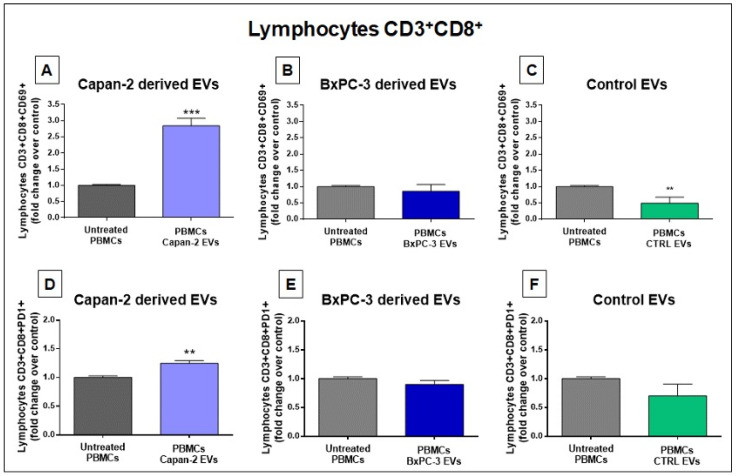
Capan-2, but not BxPC-3 or control EVs, increases the proportion of CD8+CD69+ and CD8+PD-1+ lymphocytes. The proportion of CD8+CD69+ lymphocytes is increased after 48 h treatment of PBMCs with EVs derived from Capan-2 cell line (**A**) in comparison to untreated PBMCs, whereas treatment with BXPC-3-derived EVs (**B**) did not increase the proportion of CD8+CD69+ lymphocytes. A slight decrease in the proportion of CD8+CD69+ lymphocytes was observed after treatment with heterologous control EVs (**C**). The proportion of CD8+PD-1+ lymphocytes shows an increase only after treatment with EVs derived from Capan-2 cells (**D**) but not with BXPC-3-derived EVs (**E**) or heterologous control EVs (**F**). The graphed results are the mean + SD of at least three determinations, using PBMCs and heterologous EVs isolated from two different healthy donors. * Statistically significant differences between untreated PBMCs and EV-treated PBMCs (** *p* < 0.01, *** *p* < 0.001).

The analysis of CD8+ in PBMCs from two healthy donors treated with EVs derived from Capan-2 cells showed that the proportion of CD3+CD8+CD69+ lymphocytes was markedly increased compared to untreated PBMCs (*p* = 0.0002) (Figure 5A). Conversely, in PBMCs from the same healthy donors treated with BXPC-3-derived EVs, we did not observe an increase in the proportion of CD3+CD8+CD69+ lymphocytes (Figure 5B). Interestingly, after treatment of PBMCs from the same healthy donors with heterologous control EVs, we observed, if anything, a slight decrease in the proportion of CD3+CD8+CD69+ lymphocytes (*p* = 0.0058) (Figure 5C). The results of flow cytometry analysis using Programmed Death-1 (PD-1/CD279) in CD8+ lymphocytes were consistent with those obtained with CD69. Namely, the proportion of CD3+CD8+PD-1+ lymphocytes was increased only after PBMC treatment with EVs derived from Capan-2 cells (*p* = 0.0016) (Figure 5D) but not after treatment with EVs derived from BxPC-3 cells (Figure 5E). Moreover, the treatment of healthy donor PBMCs with heterologous EVs derived from unmatched healthy donors did not significantly modify the proportion of CD8+PD-1+ lymphocytes in comparison to untreated PBMCs (Figure 5F). Overall, the results obtained with CD69 and PD-1 markers in CD4+ and CD8+ lymphocytes are concordant and are in line with the early activation of these cells after a 48 h treatment only with Capan-2-derived EVs but not with EVs from BxPC-3 or heterologous control EVs.

### 3.4. CD3+ Lymphocyte Proteomics in Response to Treatment with Capan-2 and Control EVs

Since EVs from Capan-2 promoted the expression of lymphocyte activation markers on CD3+CD4+ and CD3+CD8+, we analyzed the proteomics of CD3+ lymphocytes sorted from PBMCs with or without treatment with Capan-2 EVs or heterologous control EVs. For this purpose, PBMCs were isolated from the fresh whole blood of two healthy donors and treated or untreated with Capan-2 EVs or heterologous control EVs. After treatment for 48 h, the PBMCs were fixed and stained using an anti-CD3 antibody. Finally, CD3+ lymphocytes were isolated by fluorescence-activated cell sorting. Then, sorted CD3+ lymphocytes were employed for the proteomic analysis. The proteins identified in CD3+ lymphocytes sorted from PBMCs treated with Capan-2-derived EVs are reported in Supplementary Table S6.

**Figure 6:**
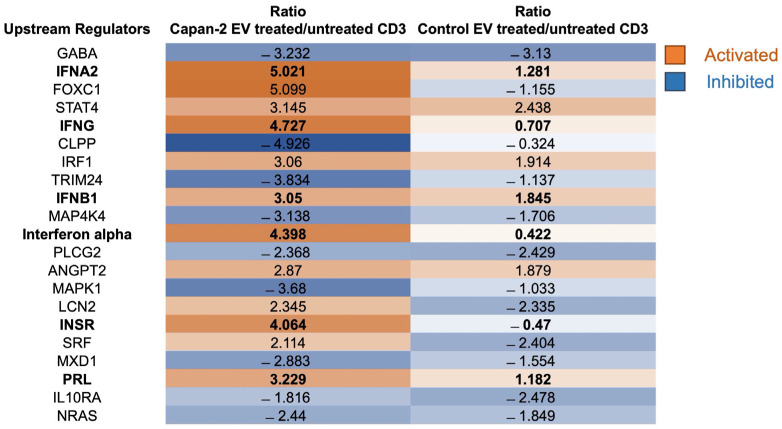
Upstream regulators analysis by IPA. The figure reports the main upstream effects highlighted by functional proteomic analysis induced in CD3 by treatment of PBMCs with Capan-2-derived EVs (Capan-2-EV-treated/untreated CD3) versus those induced by heterologous control EVs derived from CD3+ isolated from healthy donors (Control-EV-treated/untreated CD3). The box color is directly proportional to z-score values (orange for activation and blue for inhibition). z-score values > 2 or <−2 are considered statistically significant for activation or inhibition, respectively.

Upstream regulators—Upstream regulators were identified by IPA functional proteomic analysis in CD3+ sorted from PBMCs treated or untreated with Capan-2-derived EVs versus those treated or untreated with control EVs from heterologous healthy donors (Figure 6). Interestingly, most upstream regulators were immunomodulatory and significantly activated only by Capan-2 EVs. Among these, “Interferon α” (z-score 4.398), “Interferon α2” (z-score 5.021), “Interferon β1” (z-score 3.05), “Interferon γ” (z-score 4.727), “Prolactin” (z-score 3.229), and “Insulin Receptor” (z-score 4.064) were involved in the activation of immune responses. Specifically, IPA indicated as upstream regulators some molecules belonging to the type I interferon family, such as IFNα, IFNα2, and IFNβ1, and the type II interferon, such as interferon-γ (IFNG). IFNα and α2 have the potential to directly enhance the responsiveness of CD8+ T lymphocytes to specific antigens, increase their cytotoxic activity, and prolong their survival [36,37]. In particular, for interferon α and α2, the ratio of activation induced by Capan-2 EVs was, respectively, 10.4- and 3.9-fold higher than that induced by control EVs. Also, interferon-γ (IFNG) plays a key role in the activation of cellular immunity and, subsequently, in the stimulation of antitumor immune responses [38]. This molecule was one of the most activated upstream regulators in CD3+ lymphocytes after the treatment of PBMCs with Capan-2 EVs, with a ratio of activation more than 6-fold higher than that observed with control EVs (Figure 6). Among other molecules known to be involved in the activation of immune responses, the insulin receptor (INSR) is considered a marker of activation, as its expression increases upon T-cell receptor (TCR) stimulation [39]. Its ratio of activation with Capan-2 EVs was more than 8-fold higher compared to that observed with control EVs (Figure 6). In addition, prolactin (PRL) had a ratio of activation almost 3-fold higher with Capan-2 EVs compared to the results observed with control EVs (Figure 6), and this molecule was shown to increase the cytotoxic activity of T lymphocytes, as well as the secretion of proinflammatory cytokines, particularly those belonging to the type I interferon family [40].

**Figure 7:**
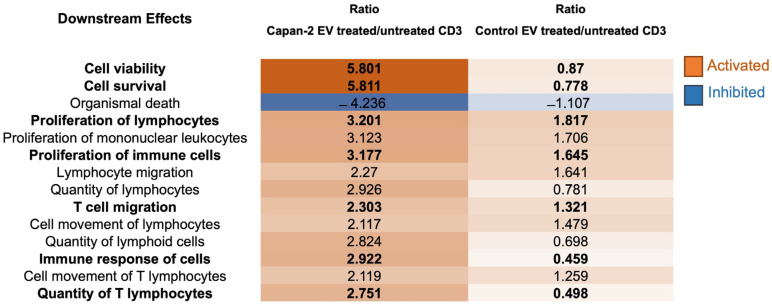
Downstream effect analysis by IPA. The table reports the main downstream effects highlighted by functional proteomic analysis induced in CD3 by Capan-2-derived EVs (Capan-2-EV-treated/untreated CD3) versus those induced by heterologous control EVs (Control-EV-treated/untreated CD3). The box color is directly proportional to z-score values (orange for activation and blue for inhibition). z-score values > 2 or <−2 are considered statistically significant for activation or inhibition, respectively.

Downstream effects—The downstream effects were identified by the IPA functional proteomic analysis of CD3+ sorted from PBMCs treated or untreated with Capan-2-derived EVs versus those treated or untreated with control EVs from heterologous healthy donors (Figure 7). Most downstream effects were activated in CD3+ lymphocytes only with Capan-2 EVs, including “Cell viability” (z-score 5.801) and “Cell survival” (z-score 5.811). In particular, for “Cell viability” and “Cell survival”, the ratios of activation induced in CD3+ lymphocytes by Capan-2 EVs were, respectively, 6.7- and 7.5-fold higher than that induced by control EVs. Most downstream effects were immunomodulatory and activated only with Capan-2 EVs, including “Proliferation of lymphocytes” (z-score 3.20), “Proliferation of immune cells” (z-score 3.18), “T cell migration” (z-score 2.30), “Immune response of cells” (z-score 2.92), and “Quantity of T lymphocytes” (z-score 2.75). In particular, the ratios of activation induced by Capan-2 EVs for “Immune response of cells” and “Quantity of T lymphocytes” were, respectively, 6.3- and 5.5-fold higher compared to that observed with control EVs. Overall, the results of the IPA analysis, including upstream regulators and downstream effects, are in line with the increased expression of early activation markers observed by flow cytometry in CD4+ and CD8+ lymphocytes.

### 3.5. PC-Derived EVs Stimulate IFNG Secretion in PBMCs

**Figure 8:**
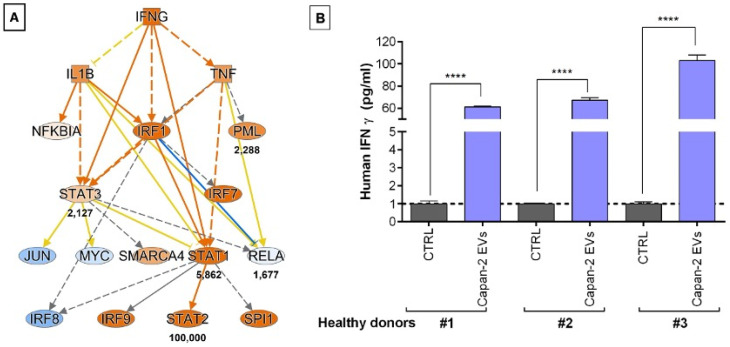
Capan-2-derived EVs increase IFNG secretion in PBMCs, as predicted by proteomic analysis. (**A**) According to upstream regulator analysis by IPA, IFNG is one of the most activated upstream regulators of CD3+ lymphocytes after treatment of PBMCs with Capan-2 EVs, and the panel shows the mechanistic network generated by IPA software, where orange nodes and edges indicate activation, while blue nodes and edges indicate inhibition. Yellow and grey reveal an inconsistent and unpredicted relationship between upstream regulators. Color intensity is directly proportional to the statistical significance of the predicted activation or inhibition. Numbers under the transcriptional regulators indicate the protein fold changes observed by LC-MS/MS analysis. (**B**) Measurement of IFNG concentration (pg/mL) by ELISA assay in supernatants of PBMCs treated or untreated (CTRLs) with Capan-2 EVs. PBMCs were isolated from 3 different healthy donors (#1, #2, and #3). IFNG concentrations were increased in supernatants of PBMCs from all three healthy donors treated with Capan-2 EVs in comparison to untreated PBMCs (**** *p* < 0.0001).

Considering that IPA predicted IFNG (Figure 6) as one of the most activated upstream regulators of CD3+ lymphocytes after the treatment of PBMCs with Capan-2 EVs, through the stimulation of a mechanistic network (Figure 8A), we analyzed by an ELISA assay the secretion of IFNG in supernatants of EV-treated or untreated PBMCs from three different healthy donors (Figure 8B). The IFNG concentrations were markedly increased in supernatants of PBMCs from all three healthy donors treated with Capan-2 in comparison to untreated PBMCs (CTRLs). These results are in line with the IPA prediction, suggesting that Capan-2-derived EVs have the potential to modulate immune responses through an increased IFNG secretion by PBMCs.

## 4. Discussion

Patients with pancreatic cancer (PC) receive limited or no benefits from radio- or chemotherapy in terms of survival, and PC remains a malignancy with one of the highest mortality rates of malignant tumors [2]. Despite great progress in immunotherapy for various cancers, PC is not sensitive to this treatment option [2]. In this regard, the presence of a highly immunosuppressive tumor microenvironment is likely to contribute to the lack of response to immunotherapy [1]. Cancer-derived EVs appear to have an immunomodulatory potential, but only a few studies have analyzed the potential role of pancreatic-cancer-derived EVs in the modulation of the immune response. Most studies focused on the immunosuppressive potential of PC-derived EVs, which may contribute to the marked immunotherapy resistance observed in PC [2]. On the other hand, EVs from colon carcinoma, mammary carcinoma, mesothelioma, mastocytoma, and chronic myeloid leukemia were shown to have an immunostimulatory potential [19]. This cancer EV property was exploited to generate antitumor immune responses, inducing T-cell-dependent eradication or growth suppression in established murine tumors [19]. With regard to pancreatic cancer EVs, their manipulation was shown to elicit immunostimulatory activity [20], but it is unknown whether PC-derived EVs in their native state may possess intrinsic immune-stimulatory potential. Investigations in this field might contribute to improving our knowledge of the immunomodulatory properties of PC-EVs, with potential implications for PC therapy. In this study, we investigated the proteomic profile of PC-derived EVs and their functional effects, analyzing their potential immunomodulatory role in the context of PBMCs. Notably, most proteins identified in our proteomic analysis of PC-EVs were included in immune-related pathways according to STRING analysis, albeit there was a substantial difference between the immune-related proteins identified in the two PC cell lines analyzed, with only approximately 50% of proteins in this pathway shared by Capan-2 and BxPC-3. Intriguingly, other differences between the protein cargo of the two PC cell lines were revealed by proteomic analysis. For example, the well-known antigenic immunostimulatory protein mesothelin was expressed only in Capan-2 EVs. Considering that there may be additional heterogeneities in protein and nucleic acid cargo not analyzed in this study, it is conceivable that these differences might have functional implications. Moreover, there were some differences in the EV size distribution between the two PC cell lines employed in our study, which, in principle, may affect cell targeting and uptake rates by the recipient cells [41] and possibly PBMCs in a different manner.

Remarkably, in our study, we observed that the effect of Capan-2 EVs on early activation markers in CD4+ and CD8+ lymphocytes was distinct from that observed with BxPC-3 EVs. Namely, an increased expression of CD69 and PD-1 early activation markers was observed only after the treatment of PBMCs with Capan-2 EVs but not after treatment with BxPC-3 EVs. In line with a specific activating effect of Capan-2 EVs on CD4+ and CD8+, an increased expression of CD69 and PD-1 early activation markers was not observed, even after the treatment of PBMCs with heterologous control EVs from different unmatched donors. The lack of CD4+ and CD8+ activation with BxPC-3 EVs and heterologous control EVs indicates that the activation observed with Capan-2 EVs was not an effect of the heterologous source of EVs employed per se. In addition, these results indicate that not all PC-EVs are able to induce early CD4+ and CD8+ activation, which may be relevant in experimental strategies aimed at inducing antitumor immune responses using cancer-EV treatment. As noted above, these distinct immunomodulatory effects might be related to differences in protein and nucleic acid cargos, as well as to the distinct size distributions of PC-derived EVs. Future studies will be necessary to unravel the potential involvement of specific proteins, nucleic acids, or distinct size distributions of PC-derived EVs in producing distinct immunomodulatory effects in PBMCs. The early lymphocyte activation observed by flow cytometry after the treatment of PBMCs with Capan-2 EVs was in agreement with the results of the proteomic analysis of CD3+ in the same experimental setting. In particular, the IPA functional proteomic analysis of CD3+ lymphocytes identified several upstream regulators associated with the activation of immune responses, such as the type I and type II interferon family, and other known activators of immune responses, such as prolactin and insulin receptors. The IPA prediction that gamma interferon was one of the top upstream regulators of CD3+ lymphocytes playing a role in response to the treatment of PBMCs with Capan-2 EVs was concordant with the ELISA results. Moreover, in support for the activation of CD4+ and CD8+ observed by flow cytometry following the treatment of PBMCs with Capan-2 EVs, the proteomic analysis indicates that, in the same experimental conditions, several downstream effects predicted by IPA in CD3+ lymphocytes were related to a marked early activation of the immune response. The observation that EVs from one of the PC cell lines analyzed may induce the activation of the immune response was somewhat surprising and at odds with the notion that PC is an immune-cold tumor. However, in a previous study, PC-EVs manipulated to deplete their miRNA cargo were shown to induce the dendritic-cell-mediated activation of CIKs, which shows that, under certain conditions, PC EVs may have immunostimulatory potential [20]. Moreover, it is known that cancer EV cargo is heterogeneous and may include immunostimulatory, as well as immunosuppressive, molecules [7]. In this study, we observed that, even without manipulation, EVs from Capan-2, one of the PC cell lines analyzed, can induce the early activation of immune responses in CD4+ and CD8+ lymphocytes and that this effect is PC-cell-line dependent since it was not observed with BxPC-3 EVs. Nevertheless, considering that one of the markers induced by Capan-2 EVs is PD-1, a marker of early activation/exhaustion, it is conceivable that the expression of this marker could be a prelude to in vivo exhaustion of the antitumor response in the long term even with EVs from this PC cell line. Considering that pancreatic cancer is one of the most immune-cold tumors, this possibility will need to be explored in future studies. Nevertheless, it is interesting that EVs from a PC cell line can activate immune responses in the short term and in the context of PBMCs that includes costimulatory cells can activate immune response. A better understanding of heterogeneous responses induced by different PC-EVs could be relevant in the design of rational EV-based antitumor vaccination protocols.

An important question with regard to in vitro immunomodulatory studies is whether the concentrations of EVs used in these studies are biologically relevant. There is variability in the EV concentrations among studies, and a ratio of 10 μg up to 200 μg of EV proteins/10^6^ T cells was used in previous studies [42]. In the present study, we used a more conservative ratio of 30 μg of EV proteins/10^6^ PBMC. Using flow cytometry, we previously showed that the concentration of intact blood-circulating EVs in patients with solid tumors, including PC, is considerably increased, reaching levels up to two-fold higher than in controls, and that cancer-derived EVs contribute, at least in part, to this sharp increase [28,43,44]. In addition, it is conceivable that the concentration of tumor-derived EVs in the proximity of tumors and draining lymph nodes may be even higher than in blood. In support of the biological relevance of EV concentrations used to investigate immunomodulation in vitro, a previous study showed that the concentration of circulating EVs in cancer patients is approximately 2 × 10^11^ EVs/mL of plasma and that 5 × 10^7^ EVs contain approximately 5 μg of EV proteins. Thus, the EV concentrations commonly used in EV-mediated immunomodulation studies, including the one used in the present study (75 μg of EV proteins/mL ≈ 10^9^ EVs/mL), are approximately 100-fold lower than those of circulating EVs in cancer patients, supporting the biological relevance of the effects observed in vitro.

## 5. Conclusions

Previous studies highlighted that pancreatic cancer EVs may exert a direct immunosuppressive role on pre-stimulated T cells [12] but also a dendritic-cell-mediated immunostimulatory effect on CIKs after the depletion of miRNA cargo, suggesting a pleiotropic potential of PC-EVs on the immune system [20]. In this study, we observed that immune-related proteins are highly enriched in pancreatic cancer EVs and that the “Immune System” represents one of the top pathways revealed by the proteomic analysis of PC EVs, supporting their key role in immunoregulation. In addition, we observed that PBMC stimulation with unmanipulated Capan-2, but not BxPC-3, PC EVs may produce immunostimulatory effects on CD4+ and CD8+ lymphocytes. We also observed that EVs from healthy donors had no immunostimulatory effect on CD4+ and CD8+ in the context of PBMCs derived from heterologous donors, further supporting the observation that the immune activation induced by Capan-2 EVs is not simply due to the heterologous source of EVs and PBMCs. These results are in line with the pleiotropic cargo of cancer EVs [7] that might facilitate either immunostimulatory or immunosuppressive interactions, at least in the short term. A better understanding of the heterogeneous effects on immunoregulation observed with EVs derived from different pancreatic cancer cell lines might be relevant for the development of highly needed immunotherapeutic strategies in this immune-cold tumor.

## Data Availability

The data that support the findings of this study are available from the corresponding author, S.V. (Serena Veschi), upon reasonable request.

## Supplementary Materials

The following supporting information can be downloaded at https://www.mdpi.com/article/10.3390/cancers16101795/s1: Original Images for Blot. Supplementary Table S1—List of reagent mix used by Flow Cytometry. Supplementary Table S2—List of the 95 and 97 proteins identified by proteomic analysis in EVs derived from Capan-2 and BxPC-3, respectively. Supplementary Table S3—List of Ingenuity Pathways Analysis (IPA) downstream pathways of functional assignments for Capan-2 EV proteins. Supplementary Table S4—Reactome Pathways identified by STRING for Capan-2- and BxPC-3-EV proteins. Supplementary Table S5—List of EV Proteins in Capan-2 (orange) vs. BxPC-3 (green) EVs identified by STRING, as involved in REACTOME immune system pathway. Supplementary Table S6—List of the 60 unique proteins identified by proteomic analysis in CD3+ lymphocytes treated with Capan-2 EVs and not present in untreated CD3.
