# Supplementary material for: Exploring the Immunomodulatory Potential of Pancreatic Cancer-Derived Extracellular Vesicles through Proteomic and Functional Analyses
Source: Cancers (Basel). 2024 May 8;16(10):1795. doi: 10.3390/cancers16101795 (PMC11120044; doi:10.3390/cancers16101795)
Supplement: Supplementary file 1 [file cancers-16-01795-s001.zip › Supplemetary Table S1.docx]

**Supplemetary Table S1: Reagent mix used by Flow Cytometry**
