# Supplementary figures and images for: Exploring the Immunomodulatory Potential of Pancreatic Cancer-Derived Extracellular Vesicles through Proteomic and Functional Analyses
Source: Cancers (Basel). 2024 May 8;16(10):1795. doi: 10.3390/cancers16101795 (PMC11120044; doi:10.3390/cancers16101795)

**Reactome Pathways identified by STRING for Capan-2 and BxPC-3 EV proteins**

**Capan-2 EVs**


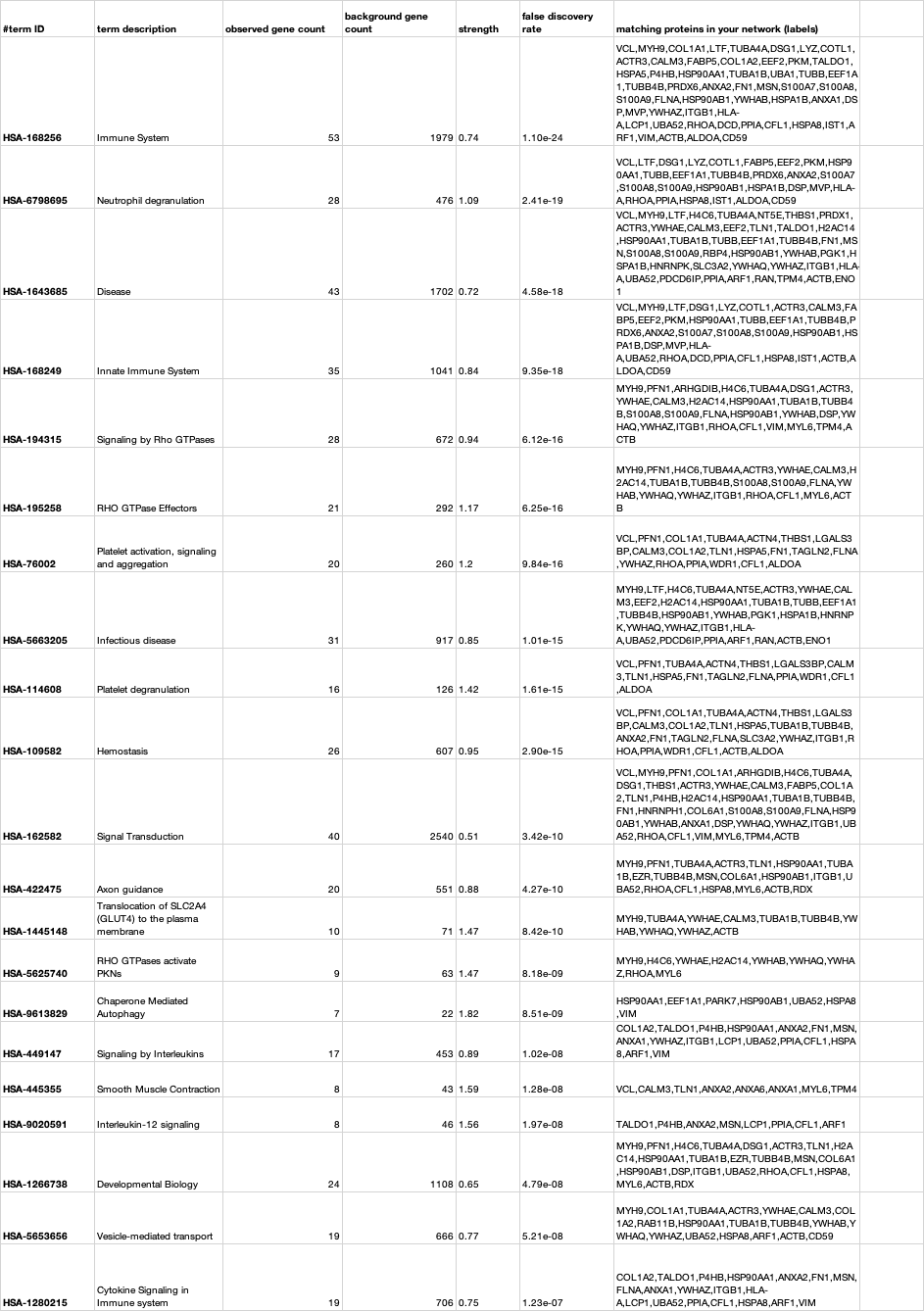


**BxPC-3 EVs**


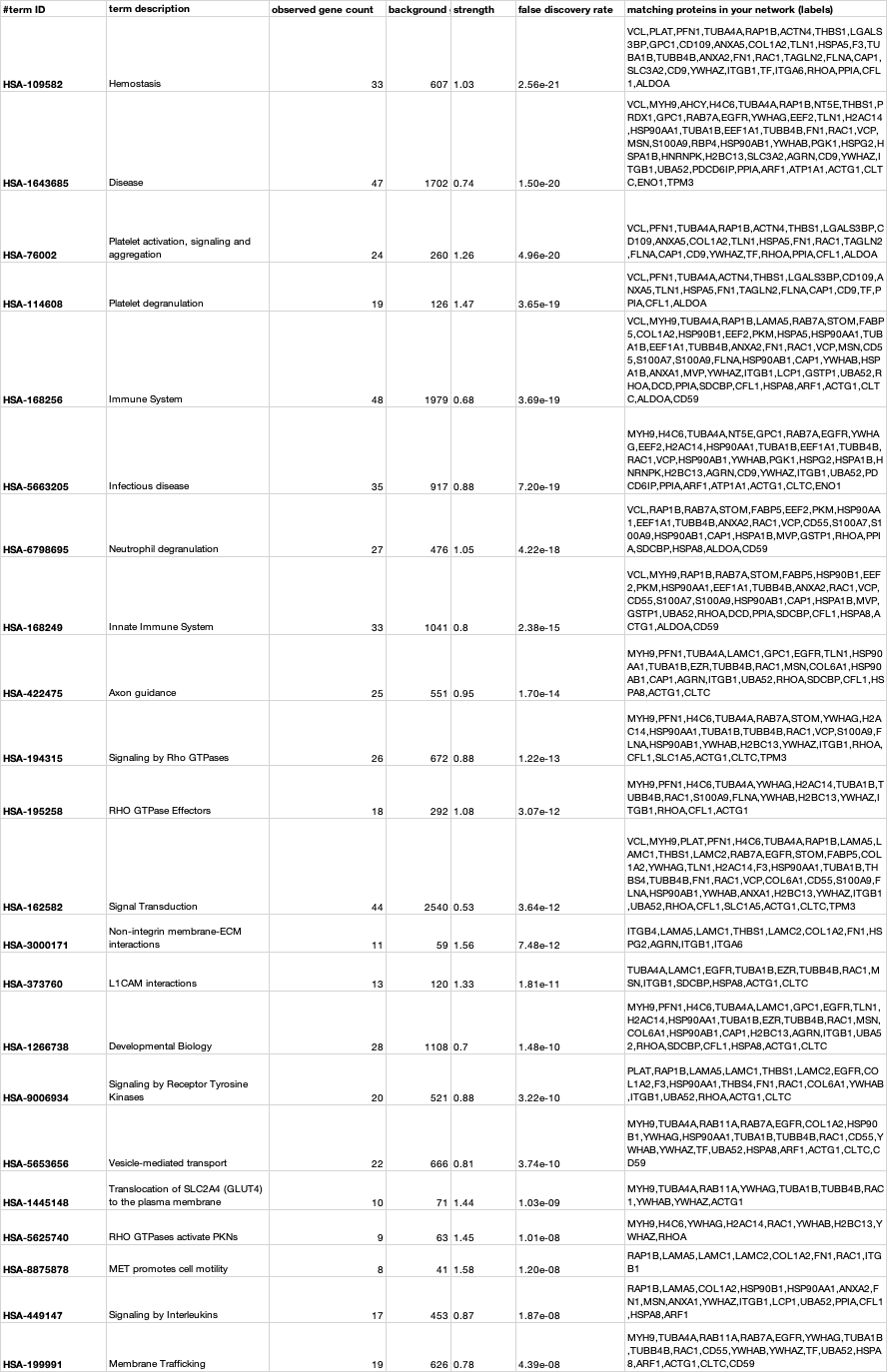

Supplement: Supplementary file 1 [file cancers-16-01795-s001.zip › Supplementary Table S4 .docx]
